# In vitro activity of clindamycin, doxycycline, and trimethoprim/sulfamethoxazole against clinical isolates of β-hemolytic *Streptococcus* spp. via BD Phoenix and broth microdilution

**DOI:** 10.1017/ash.2023.515

**Published:** 2023-12-15

**Authors:** Christian Cho, Ryan K Shields, Ellen G Kline, Thomas L. Walsh, Chelsea E. Jones, Karen Kasarda, Kelly Stefano, Matthew A. Moffa, Derek N. Bremmer

**Affiliations:** 1 Department of Pharmacy, Allegheny Health Network, Pittsburgh, PA, USA; 2 Antibiotic Management Program, University of Pittsburgh Medical Center, Pittsburgh, PA, USA; 3 Department of Medicine, University of Pittsburgh, Pittsburgh, PA, USA; 4 XDR Pathogens Laboratory, University of Pittsburgh Medical Center, Pittsburgh, PA, USA; 5 Division of Infectious Diseases, Allegheny Health Network, Pittsburgh, PA, USA; 6 Department of Pathology and Laboratory Medicine, Allegheny Health Network, Pittsburgh, PA, USA

## Abstract

We tested 85 isolates of β-hemolytic *Streptococcus* spp. against trimethoprim/sulfamethoxazole (TMP/SMX), clindamycin, and doxycycline by broth microdilution (BMD) and BD Phoenix. Susceptibility rates via BMD for TMP/SMX, clindamycin, and doxycycline were 100%, 85.5%, and 56.6%, respectively. TMP/SMX is a potential monotherapy agent for β-hemolytic *Streptococcus* skin and soft tissue infections.

## Introduction

The Infectious Diseases Society of America (IDSA) guidelines suggest that β-hemolytic *Streptococcus* spp. and *Staphylococcus aureus* are the predominant causes of skin and soft tissue infections (SSTIs).^1^ While the presence of purulence is an indication of *S. aureus*, clinicians frequently provide empiric antimicrobial therapy targeting both β-hemolytic *Streptococcus* spp. and *S. aureus*.^2^ Historically, a beta-lactam would adequately cover both, but increasing rates of community acquired methicillin-resistant *S. aureus* (MRSA) have precluded this approach. Alternatively, clindamycin has been utilized to target both pathogens, but rates of resistance have increased.^3^ Tetracyclines and trimethoprim/sulfamethoxazole (TMP/SMX) are noted to demonstrate unreliable activity against β-hemolytic *Streptococcus* spp. on the basis of several in vitro studies showing TMP/SMX resistance.^1^ These findings may be related to high thymidine content of the test media that was previously employed and is known to inhibit the activity of TMP/SMX.^4^ Since, Mueller Hinton broth has been standardized with low levels of thymidine.^5^ With this in mind, we set out to evaluate susceptibility rates of TMP/SMX, clindamycin, and doxycycline against clinical isolates of β-hemolytic *Streptococcus* spp. by automated and standard susceptibility testing methods.

## Methods

Eighty-five β-hemolytic *Streptococcus* spp. isolates were identified from January 1 2018 to June 30 2018 as part of routine workup in the microbiology department. Antimicrobial susceptibilities are not performed on β-hemolytic *Streptococcus* spp. Identification was verified utilizing matrix-assisted laser desorption ionization time of flight mass spectrometry (MALDI-TOF) on the Becton Dickinson Bruker MALDI Biotyper CA system. All isolates were stored in nutrient media at −20°C and subcultured thrice before testing on the BD Phoenix SMIC-101 *Streptococcus* panel. Of note, the BD Phoenix SMIC-101 panel is not validated for the combination of β-hemolytic *Streptococcus* spp. and TMP/SMX. These results were recorded for research purposes only and not recorded in the patients’ medical record.

Next, we determined MICs by broth microdilution (BMD), according to CLSI guidelines, for TMP/SMX (MP Biomedicals, testing range 0.06/1.14–64/1216 µg/mL), clindamycin hydrochloride (UPMC Pharmacy, 0.015–16 µg/mL), and doxycycline hyclate (MP Biomedicals, 0.008–8 µg/mL). Briefly, organisms were tested in cation adjusted Mueller Hinton broth containing 5% lysed horse blood and incubated at 35°C for 20–24 h. Isolates were tested in duplicate, triplicated if initial tests varied by greater than one doubling dilution. Quality control strain *S. pneumoniae* ATCC 49619 was used throughout. Results were interpreted according to the 2020 CLSI criteria for clindamycin and tetracycline with susceptibility breakpoints of ≤0.25 µg/mL and ≤2 µg/mL, respectively.^6^ EUCAST interpretative criteria were used for doxycycline and TMP/SMX with susceptibility breakpoints of ≤1 µg/mL and ≤1 µg/mL, respectively.^7^

Discordant results between BD Phoenix and BMD were categorized into major and very major errors, and rates of essential agreement (EA) and categorical agreement (CA) were determined using established definitions.^8^ Major errors were defined as false-resistant results, and very major errors were defined as false-susceptible results, based on current CSLI and EUCAST breakpoints as defined above.^6,7^ It should be noted that the comparison of tetracycline from BD Phoenix to doxycycline by BMD may result in some inaccuracy as they are different agents within the same class.

## Results

Of the 85 isolates tested, the most common species identified was *S. pyogenes* (*n* = 49), followed *by S. agalactiae* (*n* = 20) and *S. dysgalactiae* (*n* = 16). Using the BD Phoenix SMIC-101 *Streptococcus* panel, susceptibility rates to TMP/SMX, clindamycin, and tetracycline were reported as 44.7%, 62.4%, and 61.2%, respectively. The corresponding rates of susceptibility by BMD testing were 100%, 85.5%, and 56.6%, respectively (Table 1). The TMP/SMX, clindamycin, and doxycycline MIC_50_/MIC_90_ via BMD were 0.12/0.25 µg/mL, 0.03/>16 µg/mL, and 0.12/8 µg/mL, respectively. All beta-lactams, linezolid, and vancomycin were susceptible at 100%, while erythromycin (45.9%), levofloxacin (94.1%), and moxifloxacin (88.2%) had variable susceptibility. Not all isolates that were tested by BD Phoenix were viable for BMD testing.

**Table 1.** Susceptibility results

Broth microdilution testing		BD Phoenix panels	
	% susceptible		% susceptible (*n* = 85)
TMP/SMX (*n* = 83)^b^	100.0%	TMP/SMX^a^	44.7%
Clindamycin (*n* = 76)^b^	85.5%	Clindamycin	62.4%
Doxycycline (*n* = 83)^b^	56.6%	Tetracycline	61.2%

a Results for beta-hemolytic strep and tmp/smx are for research use only.

b Not all isolates were viable for BMD testing.

Rates of major errors for clindamycin and doxycycline testing by the BD Phoenix system were 18.4% and 0%, respectively. Very major errors were 0% and 3.6%, respectively (Table 2).

**Table 2.** Categorical and essential agreement of BD Phoenix compared to BMD

	Categorical agreement	Essential agreement	Rate of minor errors	Rate of major errors	Rate of very major errors
TMP/SMX (*n* = 83)^a,b^	45.8%	24.1%	0.0%	54.2%	0.0%
Clindamycin (*n* = 76)^b^	78.9%	61.8%	2.6%	18.4%	0.0%
Doxycycline (*n* = 83)^b^	96.4%	39.8%	0.0%	0.0%	3.6%

Note. TMP/SMX, trimethoprim/sulfamethoxazole.

a Performed for research purposes only. TMP/SMX is not FDA-approved for β-hemolytic *Streptococcus* spp.

b Not all isolates were viable for BMD testing.

There were slight differences in susceptibility based on *Streptococcus spp*. by BMD. For S. pyogenes (*n* = 49), TMP/SMX (100%) and clindamycin (91.5%) had high rates of susceptibility, but doxycycline (75.5%) was lower. Similarly, *S. dysgalactiae* (*n* = 16) had a similar trend of TMP/SMX (100%), clindamycin (92.3%), and doxycycline (57.1%). *S. agalactiae* (*n* = 20) had high TMP/SMX (100%) susceptibilities, but clindamycin (62.5%) and doxycycline (10%) were lower.

## Discussion

Our in vitro analysis found β-hemolytic *Streptococcus* spp. to be 100% susceptible to TMP/SMX by the gold standard BMD method. This data supports the possibility of using TMP/SMX monotherapy for empiric SSTI treatment targeting β-hemolytic *Streptococcus* spp. Bowen and colleagues performed a randomized controlled trial including pediatrics with impetigo who were given TMP/SMX or benzathine penicillin. There was no treatment differences between groups and of interest, 90% of patients in this trial had cultured *S. pyogenes.* ^9^ This, along with clinical and other in vitro data supporting this notion, has been sufficient for our antimicrobial stewardship team to adjust our SSTI treatment algorithm for such infections at our institution.^9^ As the IDSA guidelines suggest, tetracyclines remain an unreliable option for empiric coverage of a β-hemolytic *Streptococcus* spp. given the high rates of resistance.

In our analysis, clindamycin had an overall susceptibility rate of 85.5% by BMD. Interestingly, there was a high rate, 18.4%, of major errors indicating that the BD Phoenix reported isolates resistant when they were truly susceptible by the gold standard BMD. Regardless, we find it interesting that clindamycin was shown to be a less reliable empiric drug for the treatment of β-hemolytic *Streptococcus* spp. than TMP/SMX. There are broad clinical implications for the declining rate of clindamycin susceptibilities. One area where this may make a large impact is for surgical prophylaxis where many patients who are considered beta-lactam allergic receive clindamycin as an alternative. It is well described that patients who receive non-beta-lactam agents have higher rates of surgical site infections.^10^ This may be an even more pronounced effect if targeting *S. agalactiae*, common for obstetrics, as other published reports are consistent with our current study, reporting significant rates of clindamycin resistance in this species.^3^ It would appear that *S. agalactiae* resistance to doxycycline is also significantly more common than other β-hemolytic *Streptococcus* spp. For treatment or prophylaxis against a β-hemolytic *Streptococcus* spp., practitioners may want to refrain from using empiric clindamycin or doxycycline.

In conclusion, TMP/SMX was universally susceptible against our clinical isolates of β-hemolytic *Streptococcus* spp. using the gold standard BMD. Our study indicates that TMP/SMX is a potential monotherapy agent for the treatment of SSTIs caused by β-hemolytic *Streptococcus* spp., while clindamycin and particularly doxycycline may be less reliable empiric options.
